# Moderate Folic Acid Supplementation in Pregnant Mice Results in Altered Sex-Specific Gene Expression in Brain of Young Mice and Embryos

**DOI:** 10.3390/nu14051051

**Published:** 2022-03-02

**Authors:** Yan Luan, Marta Cosín-Tomás, Daniel Leclerc, Olga V. Malysheva, Marie A. Caudill, Rima Rozen

**Affiliations:** 1 Departments of Human Genetics and Pediatrics, McGill University, Montreal, QC H3A 0C7, Canada; yan.luan@mail.mcgill.ca (Y.L.); marta.cosin@isglobal.org (M.C.-T.); daniel.leclerc@affiliate.mcgill.ca (D.L.); 2 Research Institute of the McGill University Health Center, Montreal, QC H4A 3J1, Canada; 3 Division of Nutritional Sciences and Genomics, Cornell University, Ithaca, NY 14850, USA; ovm4@cornell.edu (O.V.M.); mac379@cornell.edu (M.A.C.)

**Keywords:** folate, brain, neurodevelopment, angiogenesis

## Abstract

Food fortification and increased vitamin intake have led to higher folic acid (FA) consumption by many pregnant women. We showed that FA-supplemented diet in pregnant mice (fivefold higher FA than the recommended level (5xFASD)) led to hyperactivity-like behavior and memory impairment in pups. Disturbed choline/methyl metabolism and altered placental gene expression were identified. The aim of this study was to examine the impact of 5xFASD on the brain at two developmental stages, postnatal day (P) 30 and embryonic day (E) 17.5. Female C57BL/6 mice were fed a control diet or 5xFASD for 1 month before mating. Diets were maintained throughout the pregnancy and lactation until P30 or during pregnancy until E17.5. The 5xFASD led to sex-specific transcription changes in P30 cerebral cortex and E17.5 cerebrum, with microarrays showing a total of 1003 and 623 changes, respectively. Enhanced mRNA degradation was observed in E17.5 cerebrum. Expression changes of genes involved in neurotransmission, neuronal growth and development, and angiogenesis were verified by qRT-PCR; 12 and 15 genes were verified at P30 and E17.5, respectively. Hippocampal collagen staining suggested decreased vessel density in FASD male embryos. This study provides insight into the mechanisms of neurobehavioral alterations and highlights potential deleterious consequences of moderate folate oversupplementation during pregnancy.

## 1. Introduction

Optimal folate status is essential for normal brain development and function due to the involvement of folate derivatives in key processes, including synthesis of nucleotides and neurotransmitters, methylation reactions, and regulation of plasma homocysteine concentrations [1,2]. Methylenetetrahydrofolate reductase (MTHFR) is an important enzyme for folate-dependent methylation reactions. It synthesizes methyltetrahydrofolate for the remethylation of homocysteine to methionine, which is then used to produce S-adenosylmethionine (SAM) for various methylation reactions. An alternate pathway to generate methionine is present, primarily in the liver and kidney, using the choline metabolite betaine as a methyl donor. Choline utilization for methylation is increased when folate metabolism is disturbed [3]. Reduced choline pools could affect the synthesis of the neurotransmitter acetylcholine and many important phospholipids [4].

The requirement for folate during pregnancy is increased to support the growth and development of both maternal and fetal tissues [5]. Because folate supplementation reduces the risk of neural tube defects [6], food fortification with folic acid (FA) has been adopted in many countries as a public health effort. FA supplements (≥400 µg/d) are also recommended during the periconceptional period. However, food fortification in combination with increased use of vitamin supplements has led to high maternal folate status in many women. Epidemiologic studies have shown that FA consumption exceeds the recommended upper limit of 1000 µg/d in 20–30% of pregnant North American women [7,8], raising concerns about potential detrimental effects. Adverse effects associated with high FA intake during pregnancy have been reported in human studies, including infant psychomotor development delay [9] and changes in cognitive development [10]. In a recent murine study [11], we found neurobehavioral abnormalities in 3-week-old pups, including hyperactivity-like behavior and short-term memory impairment, born to dams consuming FA at fivefold higher than the recommended (5xFASD) level. Downregulated hepatic MTHFR and disturbed folate and choline metabolism were identified as potential contributors. In a follow-up study to investigate the origins of the abnormal behaviors [12], we examined the impact of the same 5xFASD on embryos and placentas at embryonic day (E) 17.5. Altered methyl metabolites in the maternal plasma, placenta, and embryonic liver and changes in the placental transcriptome profile were identified [12]. Many of the metabolic and molecular changes were sex-specific. FASD altered the expression of 18 placental genes involved in angiogenesis at E17.5. Angiogenesis is essential for the development of the neural tissue and the brain vessel network.

The goal of this study was to further elucidate mechanisms by which moderate folate supplementation led to abnormal behaviors in 3-week-old pups by examining the impact of 5xFASD on brain development at two developmental stages, namely, postnatal day (P) 30 and E17.5, with an emphasis on gene expression. We hypothesized that 5xFASD would lead to transcriptome changes in both postnatal and prenatal brains, especially within the critical pathways for brain development, including genes involved in neural growth and development. As we previously found that 5xFASD affected placental angiogenesis, we hypothesized that genes related to angiogenesis could also be affected in developing brains.

## 2. Materials and Methods

### 2.1. Animals and Diets

All procedures were conducted in accordance with Canadian Council on Animal Care guidelines and approved by the RI-MUHC Animal Care Committee (AUP 3132). Mice used in this study have been described in previous publications [11,12]. Briefly, at weaning, female C57BL/6 mice were randomly fed CD (2 mg/kg FA, recommended amount for rodents) or 5xFASD (10 mg/kg FA) (Table S1) for 4 to 5 weeks. Females were then mated with C57BL/6 males. Diets were maintained throughout the pregnancy and lactation until P30 or during pregnancy until E17.5. Whole cerebral cortices of P30 pups and embryonic cerebrum were collected, snap-frozen, and stored at −80 °C. For some embryos, whole heads were collected and fixed.

### 2.2. Measurement of Methyl Metabolites

SAM and S-adenosylhomocysteine (SAH) were measured in frozen embryonic cerebrum (*n* = 7–8/group) by liquid chromatography–electrospray ionization–tandem mass spectrometry (LC–MS) as before [13,14].

### 2.3. RNA and DNA Purification

The total RNA and DNA of frozen P30 cortex and E17.5 cerebrum were extracted from CD and FASD males and females (*n* = 6–8/group) using an AllPrep DNA/RNA/miRNA Universal Kit (Qiagen) with DNase I treatment (Qiagen) as appropriate to avoid DNA contamination of RNA.

### 2.4. Microarray Analysis

Microarray analysis of gene expression was performed by McGill University and Génome Québec Innovation Centre, using mouse Affymetrix Clariom S Array (Affymetrix), and RNA from CD and FASD males and females (*n* = 4/group) of pups and embryos. RNA quality, assessed by a 2100 Bioanalyzer (Agilent), had an integrity number ≥ 8.5. Data were analyzed by Transcriptome Analysis Console software (TAC 4.0.2.15; Affymetrix) using the same parameters as before, transcripts with fold changes ≥1.5 and *p* ≤ 0.05 [12]. The complete data set has been uploaded to the Gene Expression Omnibus database at the National Center for Biotechnology Information (GEO: GSE193355 for the P30 cortex; GEO: GSE193352 for the E17.5 cerebrum).

### 2.5. Quantitative Reverse Transcriptase PCR (qRT-PCR)

cDNA synthesis and qRT-PCR were performed as before [15]. Primer sequences and conditions are described in Table S2. For the E17.5 cerebrum, *Eef2*, *Gapdh*, *Actb*, and *Tbp* (full gene names are listed in Table S3) were identified as the best combination of normalization genes by both geNorm [16] and NormFinder [17]; normalization factors were calculated with geNorm. For the P30 cortex, *Actb, B2m*, and *Gapdh* were the best combination of normalizers.

### 2.6. DNA Methylation Analysis by Bisulfite Pyrosequencing

Pyrosequencing was performed as before [18] (see Table S4 for oligonucleotide sequences).

### 2.7. Immunofluorescence for Collagen IV in Brain Sections

During embryo sacrifice, whole heads were cut with the skin and muscles removed, and then fixed in 4% paraformaldehyde (PFA) in phosphate-buffered saline (PBS) at 4 °C overnight. Brains were dissected from the skull and further fixed in the same solution at 4 °C overnight. After fixation, brains were immersed with 15% and then 30% sucrose in PBS for cryoprotection before embedding with OCT (Cryomatrix, Thermo Fisher, Waltham, MA, USA) and stored at −80 °C until use. Samples were coronally cryosectioned at 10 µm thickness, and sections with hippocampal regions were mounted according to anatomical structures. Examined sections were chosen to be at the same or similar levels between mice. Brain sections were treated with L.A.B. solution (Polysciences, Inc., Warrington, PA, USA, 24310-500) for 20 min for antigen retrieval and incubated at 4 °C overnight with collagen IV (AB769, goat, Millipore, Burlington, MA, USA; 1:40 diluted in PBS with 0.5% Triton (PBST) and 10% normal donkey serum (S30-100ML, Millipore, Burlington, MA, USA)). Sections were washed 3 times with PBST for 10 min each, followed by secondary antibody (A11058, Alexa Fluor 594, Invitrogen, Waltham, MA, USA) incubation for 2 h at room temperature. Nuclei were counterstained with 4′,6-diamidino-2-phenylindole (DAPI, Sigma D9542, St. Louis, MI, USA). Images of 3 sections per animal (5 animals per group) were taken with a Leica DMRB microscope (Leica) with a DP70 digital camera (Olympus, Shinjuku City, Tokyo, Japan). Vascular areas were analyzed using AngioTool [19] in random order, blinded to diet and sex.

### 2.8. Poly(A) Tail Detection by Extension Poly(A) Test (ePAT)

Extension-mediated poly(A)-tail detection was performed with the ePAT method [20]. Details of the ePAT approach and identification and confirmation of polyadenylation sites for genes of interest, as well as TVN controls, are presented in Figure S1 and Table S5.

### 2.9. Statistical Analysis

Brain-to-body weight ratio was analyzed using linear mixed models (MIXED procedure, SPSS Statistics version 22; IBM, Endicott, NY, USA) with diet, embryo/pup sex, and diet x embryo/pup sex interactions as fixed factors and litter as a random factor. Since one animal per litter was used for gene expression and methylation quantification, these data were analyzed by 2-factor analysis of variance (ANOVA) (with diet and sex as factors), followed by Tukey’s post hoc analysis correcting for multiple testing. Student’s *t*-test for unpaired data was used where indicated. Correlation for gene expression was conducted using Pearson’s correlation. Nested ANOVA was used for vessel area comparison (R Core Team, 2020, Vienna, Austria). ANOVA, *t*-test, and correlations were performed with GraphPad Prism software (version 9.3.0; GraphPad Software, San Diego, CA, USA). Outliers by Grubbs’ test were removed. *p* < 0.05 was considered significant; *p* < 0.08 was considered a trend.

## 3. Results

### 3.1. The 5xFASD Did Not Affect Brain Weights at E17.5 or P30

Detailed biometric data (maternal food consumption, maternal body weights, litter sizes, embryonic growth, and offspring body weights) have been published [11,12]. These results were not affected by diet.

Brain-to-body weight ratios did not show dietary differences for E17.5 embryos or for P30 pups, but there were significantly higher values in female pups compared with male pups, as expected due to lower body weights (E17.5: diet *p* = 0.752, sex *p* = 0.087, diet x sex *p* = 0.342, *n* = 16–30/group; P30: diet *p* = 0.176, sex *p* < 0.001, diet × sex *p* = 0.285, *n* = 21–25/group).

### 3.2. Sex-Dependent Impact of Maternal FASD on Cerebral Cortical Gene Expression in P30 Pups

**Figure 1:**
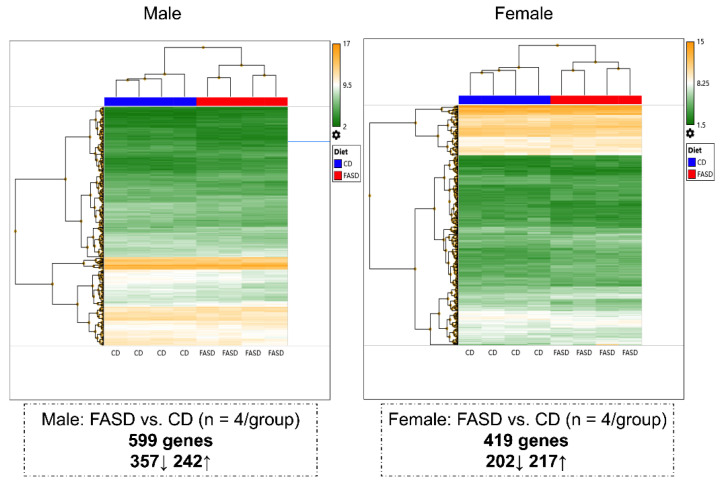
Hierarchical clustering showing different changes in P30 cortical gene expression due to maternal FASD in males and females. A total of 599 genes (357 downregulated, 242 upregulated) were differentially expressed in FASD males compared with CD males, and 419 genes (202 downregulated, 217 upregulated) were differentially expressed in FASD females compared with CD females.

Microarray analysis was conducted in cortices from male and female pups to examine gene expression changes in the brain. We first compared dietary effects using sex-segregated data. Overall, maternal FASD triggered significant expression changes in 1003 genes. However, it affected different gene sets in the two sexes with only 1.5% of the affected genes that were shared (15 genes, Table S6). A total of 599 genes (357 downregulated, 242 upregulated) were differentially expressed in FASD male pups compared with CD male pups, and 419 genes (202 downregulated, 217 upregulated) were differentially expressed in FASD female pups compared with CD female pups (Figure 1).

Hierarchical clustering was also performed to compare differences due to sex in diet-segregated data. Distinct gene expression patterns were observed in the sexes: 382 genes (202 downregulated, 180 upregulated) were differentially expressed in CD females compared with CD males, and 452 genes (184 downregulated, 268 upregulated) were differentially expressed in FASD females compared with FASD males (Figure S2). Only 23 mutual changes were observed (Table S7).

### 3.3. qRT-PCR Assessment of Candidate Gene Expression in P30 Pups

**Figure 2:**
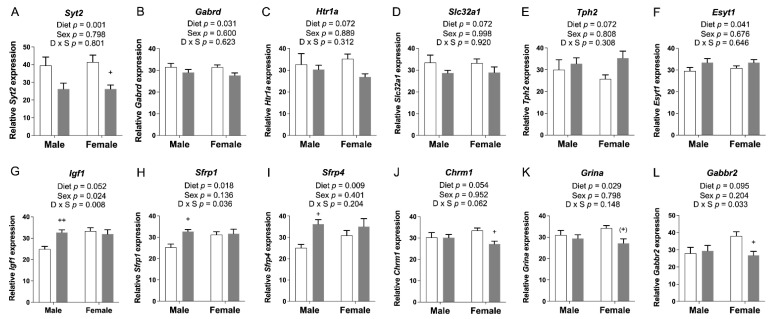
qRT-PCR assessment of candidate gene expression in P30 pup cortices. (**A**) mRNA levels of *Syt2* and (**B**) *Gabrd* were significantly decreased in FASD pups. (**C**) mRNA levels of *Htr1a* and (**D**) *Slc32a1* showed a trend toward a significant decrease in FASD pups. (**E**) mRNA levels of *Tph2* showed a trend toward a significant increase in FASD pups. (**F**) mRNA levels of *Esyt1* were significantly increased in FASD pups. (**G**) mRNA levels of *Igf1* were significantly increased in FASD male pups, and in females compared with males. (**H**) mRNA levels of *Sfrp1* and (**I**) *Sfrp4* were significantly increased in FASD male pups. (**J**) mRNA levels of *Chrm1* were significantly decreased in FASD female pups. (**K**) mRNA levels of *Grina* were significantly decreased in FASD pups, although mainly in females. (**L**) mRNA levels of *Gabbr2* were significantly decreased in FASD female pups. White bars: CD; gray bars: FASD. Values are means ± SEM of 6–8 per group. *p*-Values from 2-factor ANOVA are indicated at the top of each graph, and Tukey post hoc *p*-values are indicated as ^+^ *p* < 0.05, ^++^ *p* < 0.01, and ^(+)^ *p* = 0.056. CD: control diet; FASD: folic-acid-supplemented diet; D: diet; S: sex; D × S: diet × sex interaction.

We selected candidate genes that were involved in neuronal growth and development and synaptic transmission for confirmation by qRT-PCR. Twelve changes in expression were verified (Figure 2).

The *Syt2*, *Gabrd*, *Htr1a*, and *Slc32a1* genes exhibited a significant or a trend toward significant reduction of mRNA levels due to FASD (Figure 2A–D; full gene names are listed in Table S3). In contrast, *Tph2* and *Esyt1* were slightly increased by FASD (Figure 2E,F). These six genes are directly involved in neurotransmission: *Syt2* encodes a Ca^2+^ sensor transmembrane protein in the presynaptic terminal that is involved in the regulation of neurotransmitter fast release in central and neuromuscular synapses [21], *Gabrd* encodes the delta subunit of the GABA A receptor [22], *Htr1a* encodes a subtype of a serotonin receptor (5-HT receptor) [23], *Slc32a1* encodes an integral membrane protein involved in GABA and glycine uptake into synaptic vesicles [24], and *Tph2* encodes an enzyme essential in the biosynthesis of serotonin [25]. The increase in *Tph2* may constitute a mechanism to compensate for the downregulation of *Htr1a*. Finally, *Esyt1* encodes a lipid transfer protein involved in neurotransmission and synaptic growth [26].

Six other genes were altered by diet in one particular sex. *Igf1*, *Sfrp1*, and *Sfrp4* were upregulated in males due to FASD (post hoc FASD vs. CD males, *Igf1*: *p* = 0.009; *Sfrp1*: *p* = 0.013; *Sfrp4*: *p* = 0.039) (Figure 2G–I). Regarding sex effects, there was a significant increase in *Igf1* in females compared with males (Figure 2G). IGF1 is an important growth factor involved in mediating neuronal growth and development [27], whereas SFRPs are inhibitors of the Wnt pathway and play an essential role in cell survival, synaptic plasticity, neurotransmitter release, and presynaptic remodeling [28].

In females, *Chrm1*, *Grina*, and *Gabbr2* were downregulated by FASD (post hoc FASD vs. CD females, *Chrm1*: *p* = 0.043; *Grina*: *p* = 0.056; *Gabbr2*: *p* = 0.042) (Figure 2J–L). Remarkably, all three genes correspond to receptors of different neurotransmitter systems: CHRM1, a G protein-coupled receptor, is the most abundant acetylcholine muscarinic receptor throughout the brain [29]. GRINA corresponds to the subunit of a glutamate ionotropic NMDA receptor [30], and GABBR2 is a G protein-coupled receptor subunit of the GABA B receptor [31].

### 3.4. DNA Methylation Changes Due to FASD in the Promoters of Gabbr2 and Syt2 in P30 Pup Cortex

**Figure 3:**
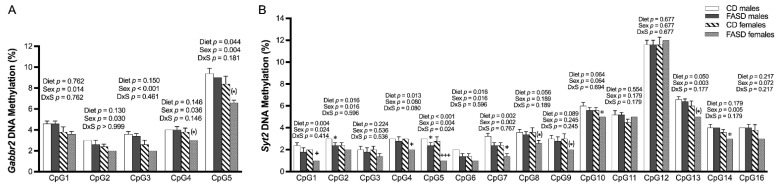
DNA methylation changes at CpGs in promoters of *Gabbr2* and *Syt2* in P30 pup cortices. (**A**) In the *Gabbr2* promoter, there was a decrease in methylation at CpG 5 due to FASD, and a general decrease in methylation in all explored CpGs in females compared with males. (**B**) In the *Syt2* promoter, there was a significant or a trend toward significant decrease in methylation at CpGs 1, 2, 4, 5, 6, 7, 8, 10, and 13 due to FASD, and a decrease or a trend for a decrease in methylation at CpGs 1, 2, 5, 6, 7, 10, 13, 14, and 16 in females compared with males. Valid data are not available for CpG15. Values are means ± SEM of 5 per group. *p*-Values from 2-factor ANOVA are indicated at the top of each CpG; Tukey post hoc *p*-values are indicated as ^+^ *p* < 0.05, ^+++^ *p* < 0.001, and unpaired *t*-test values are indicated as * *p* < 0.05, ^(^*^)^ *p* = 0.065 (*Gabbr2* CpG 4), ^(^*^)^ *p* = 0.052 (*Gabbr2* CpG 5), ^(^*^)^ *p* = 0.066 (*Syt2* CpG 8), ^(^*^)^ *p* = 0.056 (*Syt2* CpG 9), and ^(^*^)^ *p* = 0.056 (*Syt2* CpG 13). CD: control diet; FASD: folic-acid-supplemented diet; D: diet; S: sex; D × S: diet × sex interaction.

We did not observe changes due to diet in SAM or SAH concentrations in these pup brains in our previous report [11]. However, since folate is required for methylation, we explored the possibility that changes in gene expression for some of the aforementioned genes could be explained by changes in DNA methylation in their promoter regions. Using bisulfite pyrosequencing, we observed a significant decrease in methylation due to FASD at 1 out of the 5 explored CpGs (CpG5: diet, *p* = 0.044), in a CpG island in the promoter of *Gabbr2* (Figure 3A and Figure S3A). The other 4 CpGs followed the same trend (Figure 3A) but did not reach significance. Interestingly, all 5 CpGs showed methylation differences due to sex with a lower level in females compared with males.

We also observed a significant decrease in methylation by FASD at 7 CpGs out of the 15 studied CpGs in a CpG island of the *Syt2* promoter (CpG1: diet, *p* = 0.004; CpG2: diet, *p* = 0.016; CpG4: diet, *p* = 0.013; CpG5: diet, *p* < 0.001; CpG6: diet, *p* = 0.016; CpG7: diet, *p* = 0.002; CpG13: diet, *p* = 0.050) and a trend toward significance in 2 other CpGs (CpG8: diet, *p* = 0.056; CpG10: diet, *p* = 0.064) (Figure 3B and Figure S3B). The decrease in methylation due to diet was primarily in females.

We did not observe significant changes in DNA methylation in a set of CpGs located at the promoters of *Chrm1*, *Igf1*, *Sfrp1*, and *Sfrp4* genes (Table S3 and data not shown).

### 3.5. Sex-Dependent Impact of Maternal FASD on Cerebral Gene Expression in E17.5 Embryos

**Figure 4:**
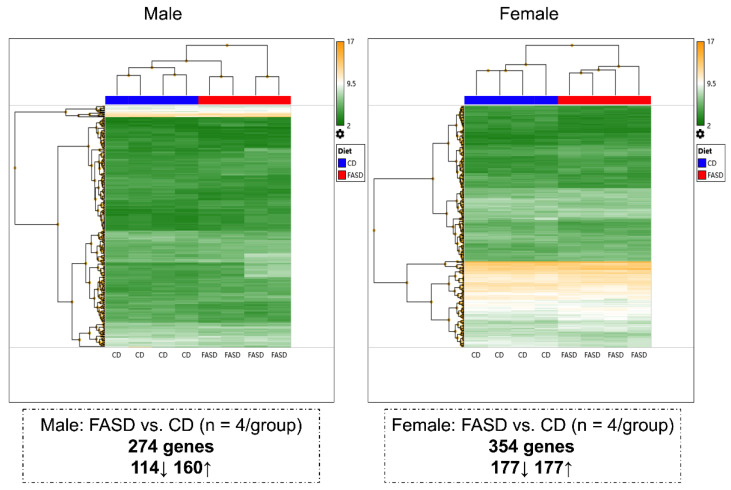
Hierarchical clustering showing different changes in E17.5 cerebral gene expression due to maternal FASD in males and females. A total of 274 genes (114 downregulated, 160 upregulated) were differentially expressed in FASD males compared with CD males, and 354 genes (177 downregulated, 177 upregulated) were differentially expressed in FASD females compared with CD females.

Microarray analysis data were compared separately in males and females to study dietary effects (Figure 4). A total of 623 expression changes were identified. A total of 274 genes (114 downregulated, 160 upregulated) were differentially expressed in FASD males compared with CD males, and 354 genes (177 downregulated, 177 upregulated) were differentially expressed when FASD females were compared with CD females. There were only 5 commonly changed genes in the two sexes (Table S8; about 0.8% of all affected genes), indicating that FASD impacts embryonic cerebral gene expression differently in males and females. Interestingly, when comparing differentially expressed genes due to diet from the pup cortex and embryonic cerebrum, only 3 genes and 12 genes were in common in males and females, respectively (Tables S9 and S10), indicating that FASD affects brain transcriptome differently at different developmental stages.

Hierarchical clustering was also performed to compare differences due to sex in the CD group and in the FASD group. The mice segregated as two sex-based main clusters and distinct gene expression patterns were observed in the two sexes: 268 genes (109 downregulated, 159 upregulated) were differentially expressed in females compared with males in CD, and 428 genes (209 downregulated, 219 upregulated) were differentially expressed in females compared with males in FASD (Figure S4). Fifteen mutual changes were observed (Table S11).

### 3.6. The 5xFASD Led to Increased Embryonic Cerebral RNA Degradation

**Figure 5:**
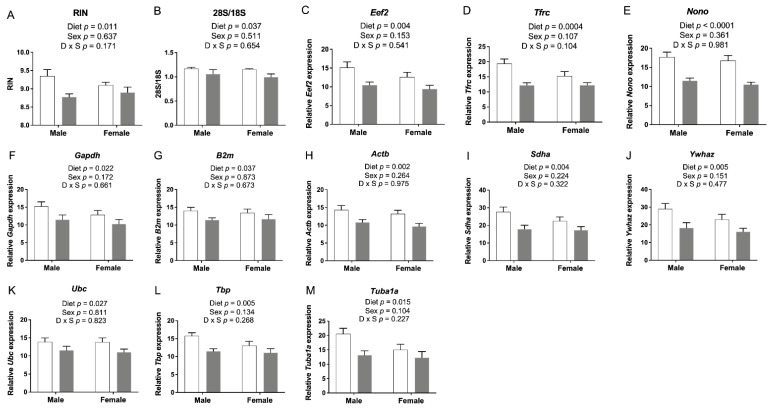
E17.5 cerebral RNA integrity and housekeeping gene expression. (**A**) RNA integrity number (RIN) and (**B**) 28S to 18S ribosomal RNA ratios were lower in the FASD group. (**C**–**M**) mRNA levels of 11 reference genes: *Eef2*, *Tfrc*, *Nono*, *Gapdh*, *B2m*, *Actb*, *Sdha*, *Ywhaz*, *Ubc*, *Tbp*, and *Tuba1a*. Values are means ± SEM of 5–6 per group. White bars: CD groups; gray bars: FASD groups. *p*-Values from 2-factor ANOVA are indicated in each graph. CD, control diet; FASD, folic-acid-supplemented diet; D: diet; S: sex; D × S: diet × sex interaction.

All embryonic cerebral RNA samples passed quality control assays using an Agilent Bioanalyzer and/or denaturing gel RNA analysis (data not shown), but interestingly, we found a significantly decreased RNA integrity number (RIN) (Figure 5A) and a decreased 28S to 18S ribosomal RNA ratio when the FASD group was compared with CD (Figure 5B). RNA samples were prepared in random order, not by dietary groups. Raw expression data for 11 tested reference (housekeeping) genes also showed a similar pattern with decreased levels in the FASD group (*Eef2*, *Tfrc*, *Nono*, *Gapdh*, *B2m*, *Actb*, *Sdha*, *Ywhaz*, *Ubc*, *Tbp*, *Tuba1a*) as shown in Figure 5C–M, respectively. These results led us to hypothesize that FASD led to increased mRNA degradation compared with CD.

**Figure 6:**
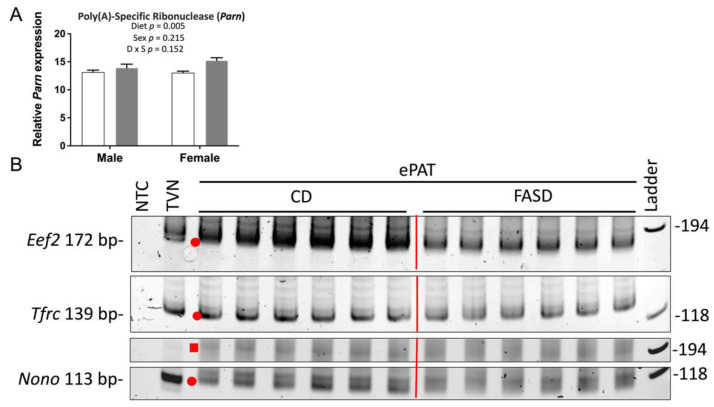
Expression of poly(A)-specific ribonuclease and ePAT reaction products of *Eef2*, *Tfrc*, and *Nono*. (**A**) mRNA levels of poly(A)-specific ribonuclease (*Parn*) were significantly increased by FASD. Values are means ± SEM of 6 per group. White bars: CD groups; gray bars: FASD groups. *p*-Values from 2-factor ANOVA are indicated in the graph. (**B**) Visualization of representative PCR products of ePAT assay (*n* = 6/group) for *Eef2*, *Tfrc*, and *Nono* on the polyacrylamide gel. Red dots and square indicate the main ePAT products for each gene in the vicinity of the TVN amplicon or above, respectively. CD, control diet; FASD, folic-acid-supplemented diet; D: diet; S: sex; D × S: diet × sex interaction.

To study the underlying mechanism by which FASD caused increased mRNA degradation, we examined the expression of *Parn*, which degrades poly(A) tails of mRNAs [32]. Exonucleolytic degradation of the poly(A) tail is often the first step in eukaryotic mRNA decay [33]. We found that *Parn* expression was significantly higher in the FASD group (Figure 6A), and thus, we hypothesized that shortening of mRNA poly(A) tails could contribute to increased mRNA degradation by FASD in E17.5 cerebrum. To further explore this hypothesis, we selected three internal control genes with a pronounced mRNA decrease by FASD, namely, *Eef2*, *Tfrc*, and *Nono* (Figure 5C–E), for investigation by ePAT, which detects polyadenylated mRNA species (Figure S1). For all three genes, the intensity of the main ePAT band was lower in the FASD group compared with the CD group (Figure 6B), indicating that the levels of polyadenylated mRNA were decreased in FASD compared with CD. Several ePAT bands were less distinct (more blurred) when FASD was compared with CD.

In order to study expression changes by qRT-PCR, geNorm [16] and NormFinder [17] were used to find the optimal combination of normalization genes among the aforementioned set of 11 housekeeping genes. A subset of 4 genes (*Eef2*, *Gapdh*, *Actb*, and *Tbp*) turned out to be the best combination by both methods. Since these 4 genes, and in fact all 11 tested housekeeping genes, were subject to RNA degradation, we reasoned that candidate genes showing decreased expression by qRT-PCR using these 4 normalizers might actually have greater decreases than exhibited when normalizing and vice versa for genes that showed increased expression by qRT-PCR.

### 3.7. qRT-PCR for Genes Involved in Angiogenesis in E17.5 Cerebrum

**Figure 7:**
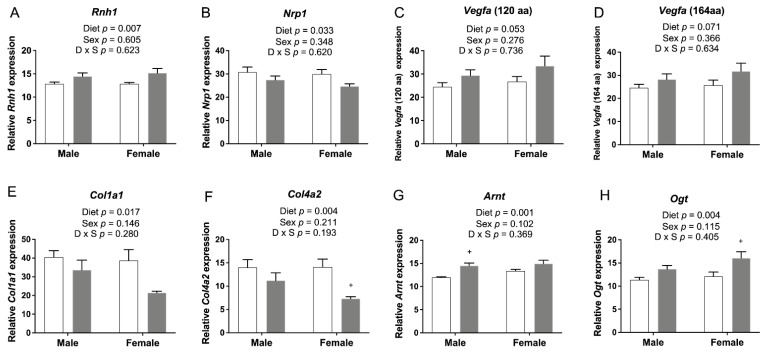
qRT-PCR of candidate genes involved in angiogenesis in embryonic cerebrum. (**A**) mRNA levels of *Rnh1* were significantly increased by FASD. (**B**) mRNA levels of *Nrp1* were significantly decreased in the FASD group. mRNA levels of two *Vegfa* isoforms, (**C**) *Vegfa* 120 and (**D**) *Vegfa* 164, showed a trend toward a significant increase by FASD. mRNA levels of (**E**) *Col1a1* and (**F**) *Col4a2* were significantly decreased in the FASD group. mRNA levels of (**G**) *Arnt* and (**H**) *Ogt* were significantly increased in the FASD group. Values are means ± SEM of 5–6 per group. White bars: CD groups; gray bars: FASD groups. *p*-Values from 2-factor ANOVA are indicated in each graph. Tukey post hoc *p*-values are indicated as ^+^ *p* < 0.05. CD, control diet; FASD, folic-acid-supplemented diet; D: diet; S: sex; D × S: diet × sex interaction.

Intensive angiogenesis occurs from the midterm of gestation until 3 weeks after birth (E8.5-10 ~ P20) in rodent brains [34]. Thus, E17.5 is an important stage for mouse brain vascular development via angiogenesis. We recently showed that FASD altered the expression of 18 placental genes known to affect the vascular network [12]. To determine whether FASD could also influence angiogenesis in embryonic brains, we used qRT-PCR to examine the expression of microarray candidate genes involved in angiogenesis and genes that were previously verified in the placenta (Figure 7). mRNA levels of *Rnh1* were significantly increased by FASD (Figure 7A). mRNA levels of *Nrp1* were significantly decreased in the FASD group (Figure 7B). RNH1 has been shown to inhibit tumor-induced angiogenesis by its interaction with angiogenin 1 [35]. NRP1 is critical for angiogenesis by functioning as a receptor for some forms of VEGF [36]. The expression of the two principal isoforms of *Vegfa* (proteins of 120 or 164 amino acids generated by alternative splicing; Figure 7C,D) showed a trend toward a significant increase by FASD. VEGF-A is a key proangiogenic factor inducing neovascularization [37]. The relative ratio of different VEGF-A isoforms has been reported to be important for their specific roles during vascular development [38]. VEGF-A_164_ binds NRP1 with high affinity [36], and the increase of *Vegfa* by FASD may be a compensatory mechanism for the decreased NRP1 coreceptor or for expression changes in other genes that disrupt angiogenesis.

The expressions of 2 genes involved in the formation of the extracellular matrix, *Col1a1* (Figure 7E) and *Col4a2* (Figure 7F), were significantly decreased by FASD. *Col1a1* encodes the pro-alpha1 chain of type I collagen, and *Col4a2* encodes a subunit for type IV collagen, a major component of the basement membrane [39]. A decreased expression of these 2 genes by FASD was previously verified in placenta [12]. Expression changes of these 6 genes may contribute to the disruption of angiogenetic pathways in the FASD group.

The expression of *Arnt* (Figure 7G) and *Ogt* (Figure 7H) was significantly increased due to FASD. Arnt encodes a subunit of hypoxia-inducible factors (HIFs), HIF-1β. Knockout of HIF-1β has been shown to result in abnormal angiogenesis and lethality in mice [40]. Increased expression in the cerebrum may be due to a compensatory mechanism. Although OGT is a placental biomarker of maternal stress and shows a protective effect against maternal insults [41], higher O-GlcNAc levels in the brain were found to be associated with defects in progenitor proliferation and premature neuronal differentiation [42]. Expression changes of these two genes by FASD were also found in the placenta; however, the expression of *Arnt* was significantly decreased by FASD, and the expression of *Ogt* was decreased in FASD males compared with CD males [12]. These different changes suggest that FASD may affect angiogenesis pathways in the placenta and embryonic cerebrum via different mechanisms.

### 3.8. The 5xFASD Resulted in Decreased Vascular Density in Male Hippocampus of E17.5 Embryos

**Figure 8:**
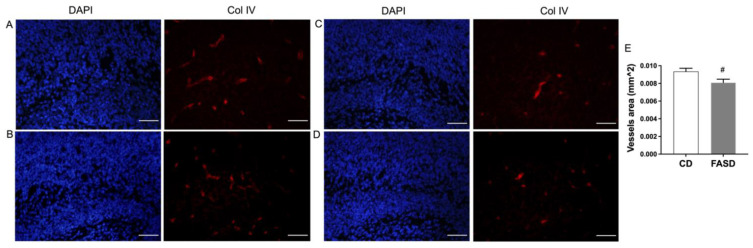
Collagen IV staining in the hippocampus of male embryos. (**A**,**B**) Representative images of two CD male embryos. (**C**,**D**) Representative images of two FASD male embryos. Left panels: DAPI staining, blue; right panels: collagen IV staining, red. (**E**) Vessel area was significantly decreased in the hippocampus of FASD males compared with CD males. Values are means ± SEM of 5 mice per group, with 3 sections per mouse. Nested ANOVA *p*-value is indicated as ^#^ *p* < 0.05. Scale bar = 100 µm.

Expression changes related to angiogenesis by FASD led us to examine the vasculature in embryonic brains. Collagen IV was used as a marker for visualizing vessels. Vessel density, measured by vessel area, was significantly decreased in the hippocampus of FASD males compared with CD males (Figure 8E). Representative images of two samples from each group are shown in Figure 8 (A,B: CD male; C,D: FASD male).

### 3.9. qRT-PCR for Genes Involved in Neurotransmission or Neuronal Growth and Development in E17.5 Cerebrum

The expression of genes involved in synaptic transmission or neuronal growth and development, which showed changes due to FASD in pups, is depicted in Figure 2. The same genes or genes involved in the same pathway were examined in the embryonic cerebrum to determine whether expression changes occurred at the earlier developmental stage.

**Figure 9:**
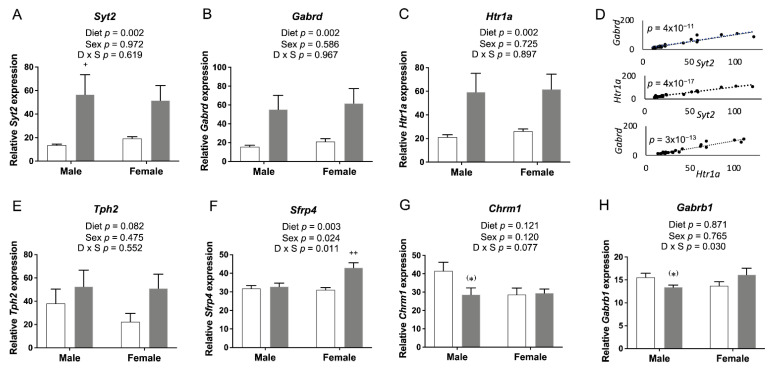
qRT-PCR of genes involved in neurotransmission and neuronal growth and development in embryonic cerebrum. mRNA levels of (**A**) *Syt2*, (**B**) *Htr1a*, and (**C**) *Gabrd* were significantly increased by FASD. (**D**) Expression of *Syt2*, *Gabrd*, and *Htr1a* showed positive correlations with each other: *Syt2* and *Gabrd*: *r* = 0.929, *p* = 4 × 10^−11^; *Syt2* and *Htr1a*: *r* = 0.976, *p* = 4 × 10^−17^; *Htr1a* and *Gabrd*: *r* = 0.946, *p* = 3 × 10^−13^. (**E**) mRNA levels of *Tph2* showed a trend toward a significant increase in the FASD group. (**F**) mRNA levels of *Sfrp4* were significantly increased in the FASD group, especially in FASD females compared with CD females. mRNA levels of (**G**) *Chrm1* and (**H**) *Gabrb1* showed a trend toward significant decreases in FASD males compared with CD males. Values are means ± SEM of 5–6 per group. White bars: CD groups; gray bars: FASD groups. *p*-Values from 2-factor ANOVA are indicated in each graph. Tukey post hoc *p*-values are indicated as ^+^ *p* < 0.05, ^++^ *p* < 0.01, and unpaired *t*-test *p*-values are indicated as ^(^*^)^ *p* = 0.059. CD, control diet; FASD, folic-acid-supplemented diet; D: diet; S: sex; D × S: diet × sex interaction.

The expression of *Syt2*, *Gabrd*, and *Htr1a* was significantly increased by FASD in the embryonic cerebrum (Figure 9A–C), while their expression was significantly decreased in FASD P30 pups (Figure 2A–C). mRNA levels of these three genes at E17.5 showed positive correlations with each other: *Syt2* and *Gabrd*: *r* = 0.929, *p* = 4 × 10^−11^; *Syt2* and *Htr1a*: *r* = 0.976, *p* = 4 × 10^−17^; *Htr1a* and *Gabrd*: *r* = 0.946, *p* = 3 × 10^−13^ (Figure 9D). The expression of *Tph2* (Figure 9E) showed a trend toward a significant increase by FASD. The expression of *Sfrp4* (Figure 9F) was significantly increased by FASD, especially in FASD females compared with CD females (post hoc *p* = 0.002), while significant increases were found in males of the FASD group compared with the CD group in P30 pups (Figure 2I). The expression of *Chrm1* (Figure 9G) showed a trend toward a significant decrease in FASD males compared with CD males in embryos (unpaired *t*-test *p* = 0.059), while a decrease was observed in females by FASD in pups (Figure 2J). The expression of *Gabrb1* (Figure 9H) showed a trend toward a significant decrease in FASD males (unpaired *t*-test *p* = 0.059).

### 3.10. DNA Methylation Changes Due to FASD in the Promoter of Syt2 in E17.5 Cerebrum

There were no changes in SAM or SAH levels due to diet in the embryonic brain (Figure S5). However, since the expression of *Syt2* was significantly increased by FASD in the embryonic cerebrum, with a substantial difference between the two diet groups, as discussed (Figure 9A), and DNA methylation changes in selected CpG sites of *Syt2* were found in P30 pups (Figure 3B), we examined *Syt2* DNA methylation in the same CpG sites in the embryonic cerebrum.

**Figure 10:**
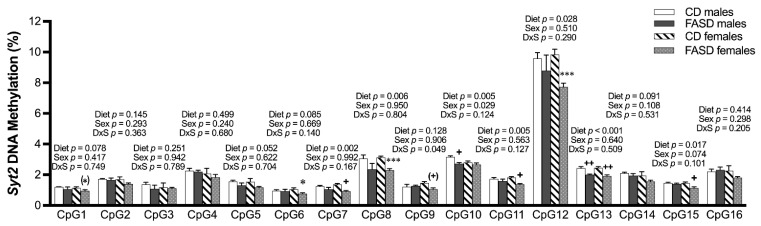
DNA methylation changes at CpGs in promoters of *Syt2* in the embryonic cerebrum. In the *Syt2* promoter, there was a significant or a trend toward significance in decreased methylation at CpGs 1, 5, 7, 8, 10, 11, 12, 13, and 15 due to FASD. There was a decrease or a trend toward a decrease in methylation at CpGs 1, 6, 7, 8, 9, 11, 12, 13, and 15 in FASD females compared with CD females, as well as a significant decrease in CpGs 10 and 13 when FASD males were compared with CD males. Values are means ± SEM of 5–6 per group. *p*-Values from 2-factor ANOVA are indicated at the top of each CpG. Tukey post hoc *p*-values are indicated as ^+^ *p* < 0.05, ^++^ *p* < 0.01, ^(+)^ *p* =0.074, and unpaired *t*-test *p*-values are indicated as * *p* < 0.05, *** *p* < 0.001, ^(^*^)^ *p* = 0.074. CD: control diet; FASD: folic-acid-supplemented diet; D: diet; S: sex; D × S: diet × sex interaction.

Significant or borderline significant overall dietary effects were observed in 9 out of 16 tested CpGs, with consistent decreases in methylation by FASD (Figure 10). As seen in pups, the decreases in methylation were more prominent in females compared with males, with 9 versus 2 significant or borderline significant changes by post hoc or unpaired *t*-test in females and males, respectively.

## 4. Discussion

In a recent study [11], we identified neurobehavioral abnormalities in P30 pups from dams fed FASD, and therefore, we embarked on an analysis of gene expression in the brains of P30 pups and E17.5 embryos. One unexpected finding was the increased RNA degradation due to FASD in E17.5 cerebrum, which was not found in E17.5 placenta or P30 cortex. In other studies from our group, we had not observed RNA degradation in other tissues using the same 5xFASD or even 10xFASD [15,43]. We are also unaware of other reports of RNA degradation following high maternal folate intake. During RNA extraction, all samples were prepared in random order, not by dietary groups, to avoid a batch effect. We found that FASD induced mRNA degradation, at least in part via shortening mRNA poly(A) tails. The increased expression of *Parn* and the decreased amount of polyadenylated mRNA of *Eef2*, *Tfrc*, and *Nono* by FASD support our hypothesis. Nonetheless, the mechanisms by which folate regulates the expression of *Parn* in a spatiotemporal manner requires further study.

Microarray analysis revealed transcription changes in the P30 cortex and E17.5 cerebrum, with 599 and 419 genes differentially expressed due to FASD in males and females for P30 pups, and 274 and 354 genes differentially expressed due to FASD in males and females for E17.5 embryos. We first chose to validate gene expression changes involved in neurotransmission or neurodevelopment in P30 pups. Most of the studied genes have been reported to be associated with neurodevelopmental diseases (NDs), which supports their contribution to the observed behavioral alterations in our previous study [11]; the changes in expression due to FASD have not been previously reported. SYT2, downregulated by FASD, is essential for survival and is downregulated in the cortex of autism spectrum disorder (ASD) [44] and schizophrenia patients [45]. Common variants in *Syt2* are associated with childhood and adult attention deficit hyperactivity disorder (ADHD) [46]. IGF1 promotes myelination and has been proposed as a potential therapy for several NDs, including ASD [47]. The increase in *Igf1* observed in FASD males may be a compensatory mechanism for the reduced sphingomyelin found in our previous study [11]. Increases in SFRPs, as observed in FASD males, are known to reduce the number of synapses in different animal models [48], and genetic and developmental studies have identified SFRP1 as a susceptibility gene for schizophrenia and ASD [49]. *Chrm1*, which was decreased in FASD female pups, is altered in the cortex of ASD [50] and schizophrenia patients [51]. The GABAergic-system-related genes *Gabbr2*, *Gabrd*, and *Slc32a1* were reduced in expression in FASD in one or both sexes. Alterations in GABA and GABA receptor expression (including downregulation of GABBR2) in different regions of ASD brains have been proposed to be involved in the etiology [52], and cortical GABAergic dysfunction is common in schizophrenia [53] and ADHD [54]. Finally, dysregulation of glutamatergic and serotoninergic genes, such as *Grina*, *Htra1*, and *Tph2*, has been reported in several NDs [54,55], and *Esyt1* is increased in the brain from schizophrenia patients [56]. Dysregulation of folate metabolism has been reported in several neuropsychiatric disorders, including ASD [57] and schizophrenia [58], but mechanisms are not fully elucidated. The impact of high folate on these disorders has received less attention, although it has been reported to influence the risk of ASD [59].

To study whether the same genes or genes in the same pathway were differentially expressed due to FASD at the earlier developmental stage, we examined their expression in E17.5 cerebrum. Consequently, the expression of six of the same genes was shown to be altered by FASD in the two groups, although not always in the same direction (*Syt2*, *Gabrb*, *Htr1a*, *Tph2*, *Sfrp4*, and *Chrm1*). For example, the expression of *Syt2*, *Gabrd*, and *Htr1a* was substantially increased in embryonic cerebrum by FASD, while their expression was decreased by FASD in the P30 cortex. These three genes showed significant correlations in expression at E17.5, suggesting coregulation by folate levels at E17.5. Interestingly, their expression has been associated with alcohol consumption. Colville et al. [60] observed that alcohol addiction was linked to differential expression of *Gabrd*, *Syt2*, and *Htr1a*. Gatta et al. [61] reported reduced GABRD expression in alcohol use disorders. Barbier et al. [62] observed that *Syt2* expression was related to gene methylation and to alcohol drinking. Since alcoholism leads to folate deficiency [63], the links between alcohol and expression of these genes could relate to changes in folate metabolism. It is also striking that the same three genes exhibit similar expression changes in Alzheimer’s disease [64]. Overexpression of these genes in embryos may hinder their normal expression at later developmental stages, when synaptic density increases and reaches adult levels by P30 [65]. Similarly, we previously found that cortical layers of E17.5 embryos appeared to be more defined due to 10xFASD, compared with CD [43], suggesting that a high FA diet may alter normal development rates.

Given the close relationship between the vasculature and nervous system during brain development at the embryonic stage and the previous findings of FASD impact on genes involved in angiogenesis in the placenta [12], we tested expression changes of several genes involved in angiogenesis in E17.5 cerebrum. The expression of RNH1, an inhibitor of the vessel growth inducer angiogenin [66], was significantly increased by FASD. The expression of NRP1, which binds to VEGF-A and fibroblast growth factors [36,67], was significantly decreased by FASD, and the increased expression of *Vegfa* may be a compensatory mechanism. The expression of *Col1a1* and *Col4a2* was significantly decreased by FASD, and immunofluorescent staining of collagen IV showed decreased vessel densities in male hippocampus due to FASD. It has been suggested that neurovascular abnormalities may contribute to the pathophysiology of NDs, such as schizophrenia [68]. The disruption of angiogenetic pathways by FASD may be a contributor to impaired neurodevelopment and altered behaviors in pups. Interestingly, RNH1, NRP1, and VEGFA also play direct roles in the nervous system. The ANG-RNH1 system is important for tRNA cleavage [69], a key process of translation repression, and for normal neurodevelopment; VEGFA has been reported to have direct effects in neurogenesis [70]; and VEGF-A_164_/NRP1-mediated neuronal migration is independent of blood vessels [71]. Some common genes in the placenta and E17.5 showed changes due to FASD but not in the same direction. The expression of *Arnt* and *Ogt* was increased by FASD in the cerebrum, but the placental expression of *Arnt* was significantly decreased by FASD and *Ogt* was significantly lower in FASD males compared with CD males, indicating different mechanisms to cope with intrauterine insult in these two tissues. We examined the expression of some of the aforementioned angiogenesis pathway genes in P30 pups by qRT-PCR but did not observe any significant differences due to diet (data not shown). Since angiogenesis during rodent brain development begins at E8.5-10 and is complete by P20, it is possible that these genes in P30 pups were not highly expressed or regulated.

Increased maternal folate intake has been reported to affect DNA methylation [72,73]. We detected minor changes in methylation for *Gabbr2* in P30 pups and *Syt2* in both P30 pups and E17.5 embryos due to FASD. Interestingly, FASD led to *Syt2* DNA methylation changes in the same direction in pups or embryos, but every CpG was not affected the same way in the two time points. Expression changes were in opposite directions. However, since the methylation levels were low for most of these CpG sites, the methylation changes may not have biological significance.

Many studies examine the impact of FA supplementation on the brain in only one sex or without specifying the sexes [74,75,76,77]. Our study investigated dietary effects for both males and females. In our recent study [11], we found differences due to diet with respect to hepatic MTHFR expression in males, with no significant changes in females, and gene expression analyses in this study showed distinct sex-segregated clusters in both the P30 cortex and the E17.5 cerebrum. qRT-PCR analysis also showed that FASD differentially affected the gene expression in males compared with females, suggesting different perturbations in the brain according to sex. This observation is consistent with the finding that complex disorders, such as NDs or neuropsychiatric diseases, present sexually dimorphic transcriptional signatures [78] with a higher risk of some disorders, such as ASD [79] and ADHD [80], in males compared with females.

The supplementation was started 4–5 weeks before mating to equilibrate the nutrient levels of the female mice to those in the diet and to ensure stable nutrient (especially folate) levels throughout the pregnancy. However, it is possible that the preconceptional exposure to higher FA could affect maternal metabolism, transcriptome, or epigenetic modifications and impact embryonic and neonatal development.

In summary, our findings suggest that moderate FA supplementation during pregnancy and lactation leads to sex-specific gene expression changes in the brain of P30 pups and E17.5 embryos, including several genes involved in angiogenesis or neurotransmission and neuronal growth and development. FA supplementation may also increase RNA degradation in the E17.5 cerebrum. These changes likely contribute to the altered behaviors reported in our previous study [11], through different mechanisms in males and females. Our work demonstrates some negative consequences of FA oversupplementation on brain development and highlights the importance of optimal folate intake during pregnancy.

## Data Availability

The complete microarray data sets have been uploaded to the Gene Expression Omnibus database at the National Center for Biotechnology Information (GEO: GSE193355 for the P30 cortex; GEO: GSE193352 for the E17.5 cerebrum).

## Supplementary Materials

The following supporting information can be downloaded at: https://www.mdpi.com/article/10.3390/nu14051051/s1: Figure S1: Extension Poly(A) Test (ePAT) methodology; Figure S2: Hierarchical clustering showing distinct sex expression pattern in P30 cortex; Figure S3: Schematic representation of CpGs analyzed by quantitative bisulfite pyrosequencing; Figure S4: Hierarchical clustering showing distinct sex expression pattern in the E17.5 cerebrum; Figure S5: Concentration of methyl metabolites in the E17.5 cerebrum; Table S1: Composition of control diet (CD) and folic-acid-supplemented diet (FASD); Table S2: Oligonucleotide primer sets used for qRT-PCR analysis (including normalizer genes) and PCR conditions; Table S3: Full gene names; Table S4: Oligonucleotide combinations used for quantitative bisulfite pyrosequencing methylation analysis of the Gabbr2 and Syt2 genes in the genomic segments described in Figure S3, as well as for the Chrm1, Igf1, Sfrp1, and Sfrp4 genes; Table S5: Oligonucleotide primer sets used for ePAT analysis of polyadenylated mRNAs; Table S6: Fifteen genes showing a diet effect in both sexes in P30 pups; Table S7: Twenty-three genes showing sex differences for both diets in P30 pups; Table S8: Five genes showing a diet effect in both sexes in E17.5 embryos; Table S9: Three genes showing a diet effect in both P30 and E17.5 males; Table S10: Twelve genes showing a diet effect in both P30 and E17.5 females; Table S11: Fifteen genes showing sex differences for both diets in E17.5 embryos.
