# Supplementary material for: Moderate Folic Acid Supplementation in Pregnant Mice Results in Altered Sex-Specific Gene Expression in Brain of Young Mice and Embryos
Source: Nutrients. 2022 Mar 2;14(5):1051. doi: 10.3390/nu14051051 (PMC8912750; doi:10.3390/nu14051051)
Supplement: Supplementary file 1 [file nutrients-14-01051-s001.zip › nutrients-1593382-supplementary.pdf]

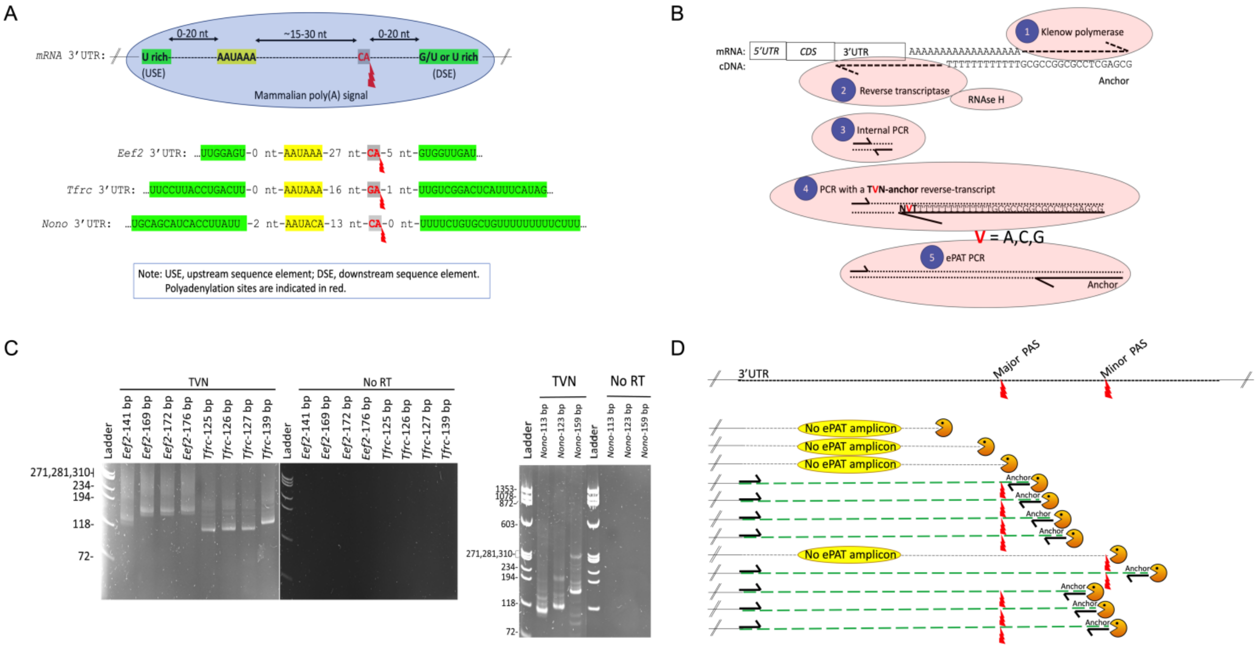

Figure S1. Extension Poly(A) Test (ePAT) methodology. (A) Consensus sequence for mammalian polyadenylation signal (PAS) [1] and specific elements in *Eef2*, *Tfrc* and *Nono* genes. We deduced the major polyadenylation sites (PAS) for these 3 genes from identification of 3’UTR uncoded adenosine stretches in NCBI cDNA databases (data not shown) and similarities to the consensus sequences. Spacing between elements of the PAS is indicated. Red thunderbolts mark poly(A) addition sites, after CA dinucleotides although other residues (as GA) can also be used [2]. (B) ePAT assesses the presence of poly(A) tails and employs a synthetic anchor sequence that anneals to the end of the poly(A) tail and provides a template for extension by Klenow. After reverse transcription and RNAse H treatment, the resulting cDNA can be used for PCR reactions. Generation of the TVN-PAT cDNA involves a different antisense anchor, with two consecutive variable bases V (A, G, or C) and N (A, C, G, T) at the 3’ end of the primer. These two bases fasten the primer to the poly(A) addition site during reverse transcription [3]. Preliminary assays were performed with the cDNAs to determine efficient sense primers that could be used in ePAT and TVN-PAT reactions (data not shown). CDS, coding DNA sequence. (C) Analysis with several

different sense oligonucleotides for TVN-PAT assays confirmed the location of the main polyadenylation site for genes that were assessed by ePAT. No amplicon was observed when reverse-transcriptase was omitted. (D) cDNA species missing a significant poly(A) tail are not detected by ePAT. Degradation of poly(A) tails may result in decreased intensity of bands as well as appearance of smears.

References

1. Proudfoot, N.J. Ending the message: poly(A) signals then and now. *Genes Dev* **2011**, *25*, 1770-1782, doi:10.1101/gad.17268411.
2. Sheets, M.D.; Ogg, S.C.; Wickens, M.P. Point mutations in AAUAAA and the poly (A) addition site: effects on the accuracy and efficiency of cleavage and polyadenylation in vitro. *Nucleic Acids Res* **1990**, *18*, 5799-5805, doi:10.1093/nar/18.19.5799.
3. Beilharz, T.H.; Preiss, T. Transcriptome-wide measurement of mRNA polyadenylation state. *Methods* **2009**, *48*, 294-300, doi:10.1016/j.ymeth.2009.02.003.

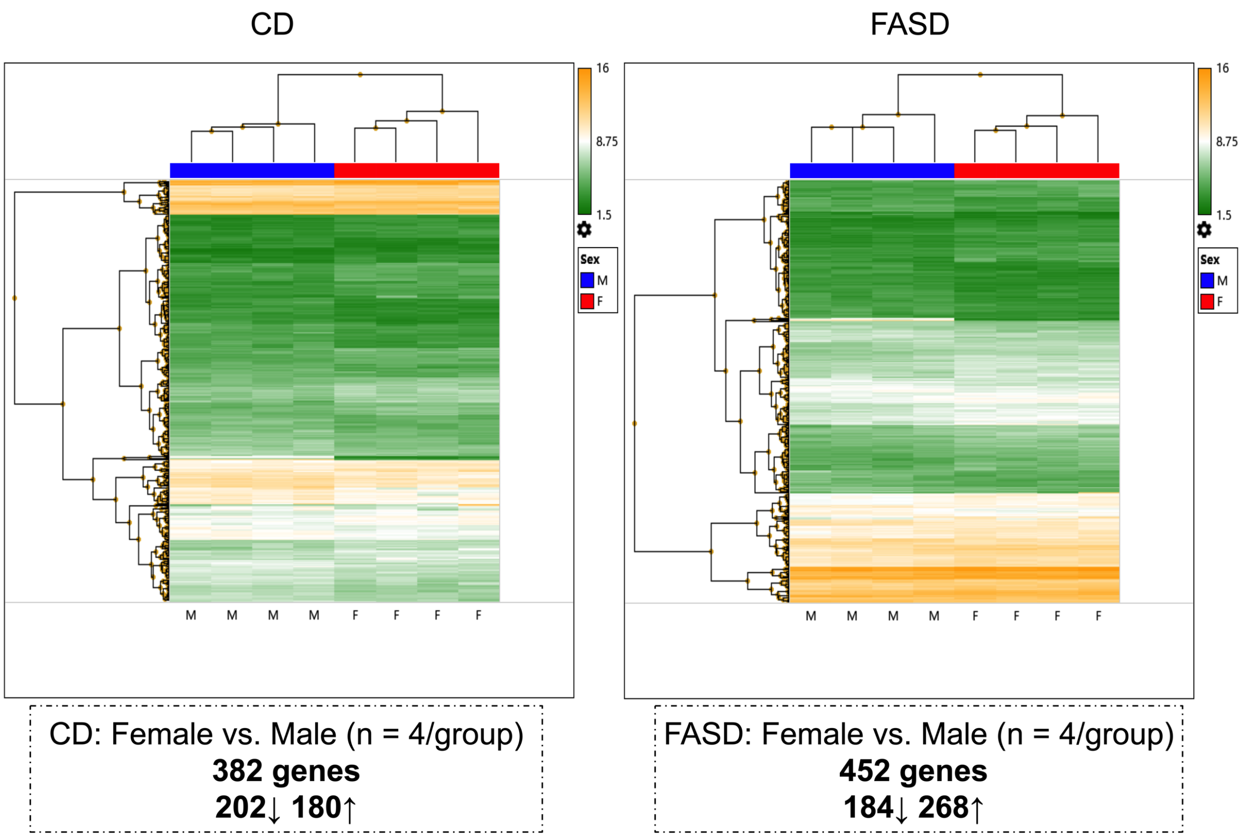

Figure S2. Hierarchical clustering showing distinct sex expression pattern in P30 cortex. 382 genes (202 down-regulated, 180 up-regulated) were differentially expressed in CD females compared to CD males, and 452 genes (184 down-regulated, 268 up-regulated) were differentially expressed in FASD females compared to FASD males.

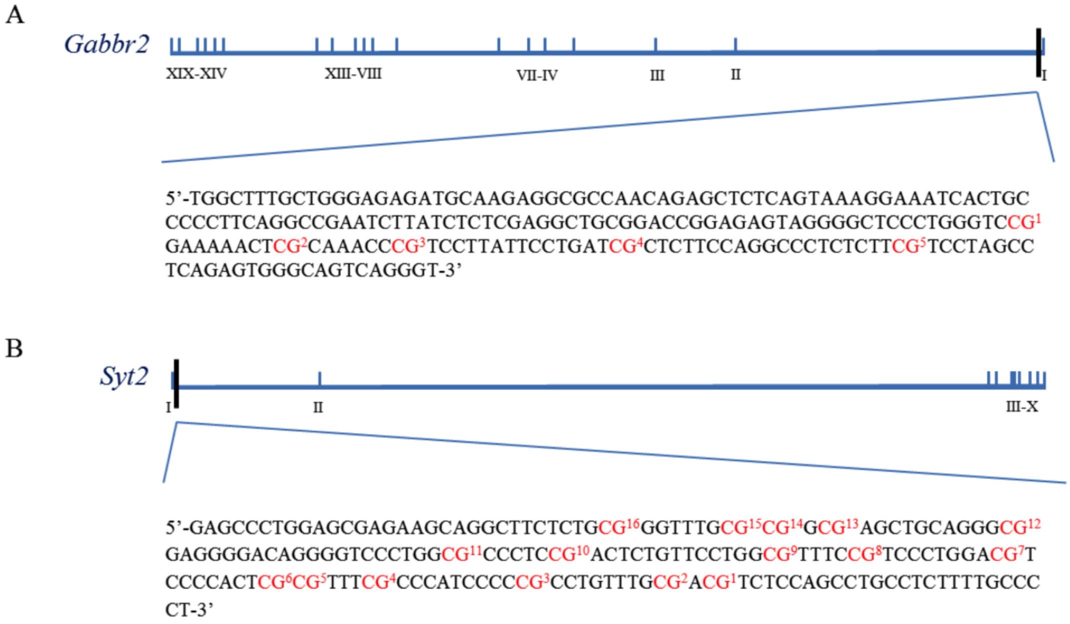

Figure S3. Schematic representation of CpGs analyzed by quantitative bisulfite pyrosequencing. (A) CpGs 1 to 5 of *Gabbr2* are included between positions 46990041-46990248 of genome assembly GRCm39 (mm39) from Genome Reference Consortium. Numbering of CpGs was arbitrarily assigned and matches the numbering in Figure 3A. (B) CpGs 1 to 16 are included between positions 134574702-134574775 of genome assembly GRCm39 (mm39) from Genome Reference Consortium. Numbering of CpGs was arbitrarily assigned and matches the numbering in Figure 3B and Figure 10.

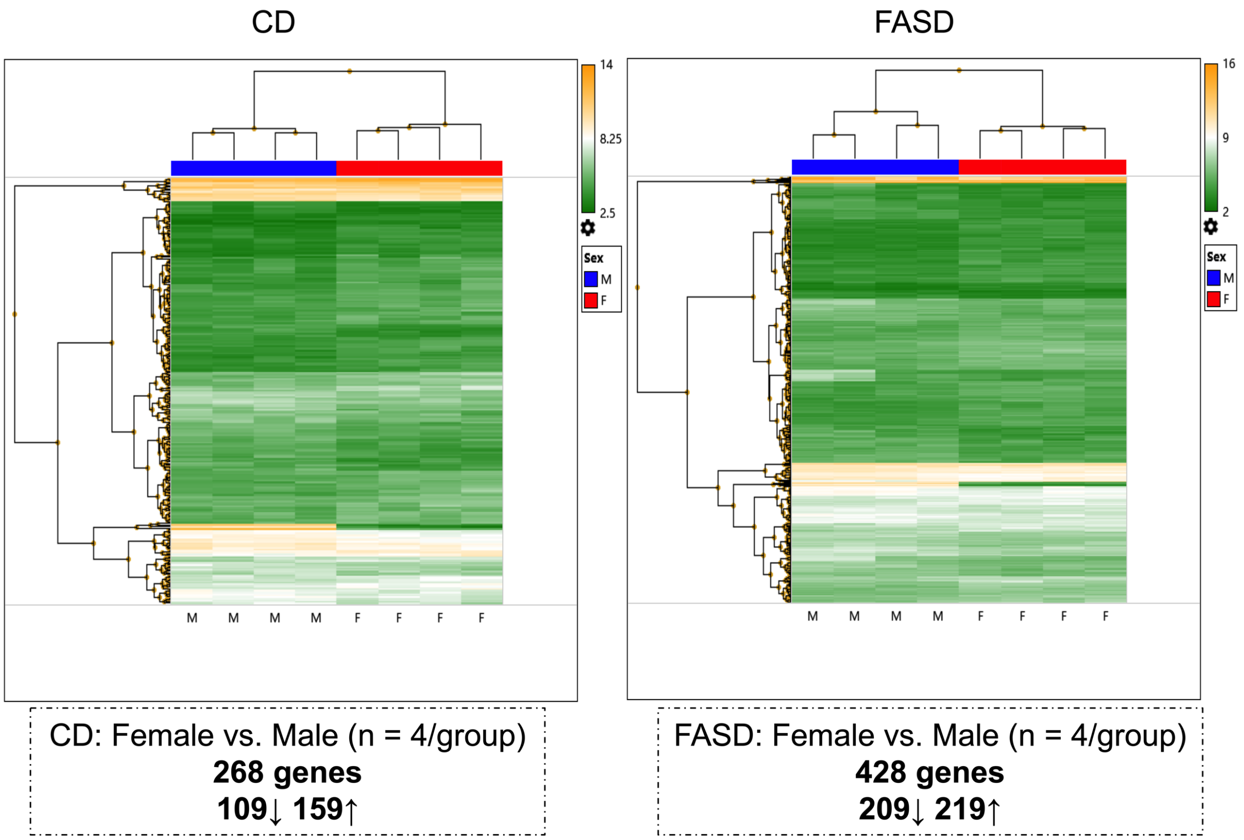

Figure S4. Hierarchical clustering showing distinct sex expression pattern in E17.5 cerebrum. 268 genes (109 down-regulated, 159 up-regulated) were differentially expressed in CD females compared to CD males, and 428 genes (209 down-regulated, 219 up-regulated) were differentially expressed in FASD females compared to FASD males.

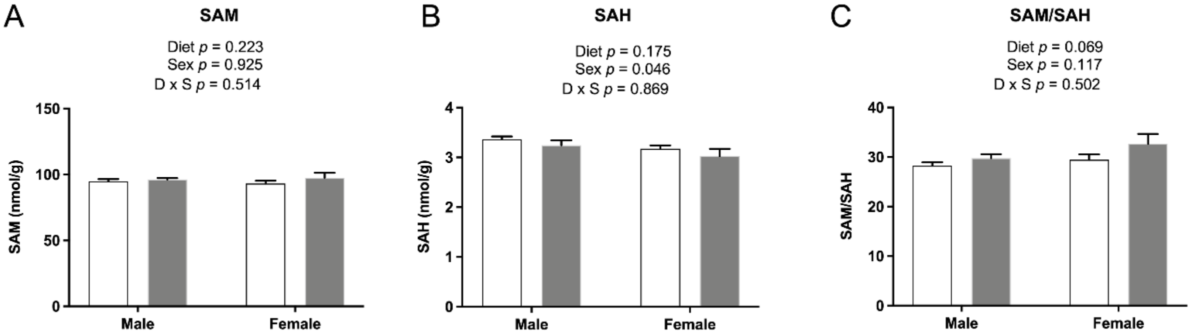

Figure S5. Concentration of methyl metabolites in E17.5 cerebrum. (A) SAM, (B) SAH, (C) SAM/SAH ratio. Values are means ± SEM of 7-8 per group. *p* values from 2-factor ANOVA are indicated at the top of each graph. CD: control diet, FASD: folic acid supplemented diet, D: diet, S: sex, D x S: diet x sex interaction. SAM, S-adenosylmethionine, SAH, S-adenosylhomocysteine.

Table S1. Composition of Control Diet (CD) and Folic Acid Supplemented Diet (FASD).

Diet Composition	CD (TD.01369) (2mg/kg Folic Acid) (g/kg)	FASD (TD.08278) (10mg/kg Folic Acid) (g/kg)
L-Alanine	3.5	3.5
L-Arginine HCl	12.1	12.1
L-Asparagine	6.0	6.0
L-Aspartic Acid	3.5	3.5
L-Cystine	3.5	3.5
L-Glutamic Acid	40.0	40.0
Glycine	23.3	23.3
L-Histidine HCl, monohydrate	4.5	4.5
L-Isoleucine	8.2	8.2
L-Leucine	11.1	11.1
L-Lysine HCl	18.0	18.0
L-Methionine	3.3	3.3
L-Phenylalanine	7.5	7.5
L-Proline	3.5	3.5
L-Serine	3.5	3.5
L-Threonine	8.2	8.2
L-Tryptophan	1.8	1.8
L-Tyrosine	5.0	5.0
L-Valine	8.2	8.2
Sucrose	349.53	349.422
Corn Starch	150.0	150.0
Maltodextrin	150.0	150.0
Soybean Oil	80.0	80.0
Cellulose	30.0	30.0
Mineral Mix, AIN-93-M-MX	35.0	35.0
Calcium Phosphate, monobasic, monohydrate	8.2	8.2
Succinylsulfathiazole	10.0	10.0
Vitamin Mix, AIN-93-VX	10.0	10.0
Choline Bitartrate	2.5	2.5
Vitamin K, menadione sodium bisulfite	0.05	0.05
tert-Butylhydroquinone (TBHQ), antioxidant	0.02	0.02
Folic Acid	-	0.008
Red food color	-	0.1

Table S2. Oligonucleotide primer sets used for qRT-PCR analysis (including normalizer genes) and PCR conditions.

Gene	Sense Primer	Antisense primer	Amplicon size (bp)	Annealing temperature (°C)	Primer molarity (μM)
Actb	CTGACGGCCAGGTCATCACTA	TAGTTTCATGGATGCCACAGGAT	105	60	0.4
Angpt2	AGAGTCCAACTACAGGATTCACC	GACCACATGCGTCAAACCAC	170	60	0.6
Arnt	TGCACAGGCTACATCAAGGC	GGGAGAACTAGTTACCTGCAGCC	126	60	0.6
B2m	ATGCTATCCAGAAAACCCCTCAA	GCGGGTGGAACTGTGTTACG	102	60	0.4
Chrm1	GGTCCCATGGAAACCCTGAAT	GCCAGCAGCCTCATTCAATC	77	60	0.4
Col1a1	CTGACGCATGGCCAAGAAGA	CCCTTGGGTCCCTCGACTC	260	60	0.6
Col4a2	CGTGACAAATACCGGGGTGA	GTCCGTAAAAACCGAGCCCT	178	60	0.6
Eef2	TGTCAGTCATCGCCCATGTG	CATCCTTGCGAGTGTCAGTGA	123	60	0.6
Esyt1	CTACCCAGGGGCATTATCCG	GGGATCCGATTTGCCCTCAA	99	60	0.4
Gabbr2	CTTCTTCGGAGTCACGGGTC	GTTGTACTCGCCGACCTTCA	109	60	0.4
Gabrb1	TCTTTTCCCGTGATGGTTGC	CGGGGGCCCTCCAAAG	141	60	0.6
Gabrd	TCAAATCGGCTGGCCAGTTCCC	GCACGGCTGCCTGGCTAATCC	147	60	0.4
Gapdh	CAGGAGCGAGACCCCACTAACAT	AAGACACCAGTAGACTCCACGAC	74	62	0.4
Grina	CCCTACCCTCAAGGAGGCTAC	CTGGCGAATGTTCTTGTCCC	192	60	0.6
Htr1a	CAACAACACCACAACGTCCC	GCTGAAGGTCACGTTGGAGA	76	60	0.4
Igf1	CTGGACCAGAGACCCTTTGC	CTCCGAATGCTGGAGCCATA	112	60	0.4
Nono	AAAGCAGGCGAAGTTTTCATTC	ATTTCCGCTAGGGTTCGTGTT	77	60	0.6
Nrp1	CAGTGGCACAGGTGATGACT	CCGTATGTCGGGAACTCTGA	108	60	0.6
Ogt	TCATCACTTTGAAAAGGCTGTC	AAGGCACGAAGATAAGCTGC	123	60	0.6
Parn	CCCATGCACTGGGTTCCAA	TCCTGCTCTCTCCTTTTGCG	156	60	0.6
Rnh1	CCAGATACAGTGCTGCGTGA	AGGCTGCTGCAACCACTATT	68	60	0.6
Sdha	GCTGTGGCCCTGAGAAAGATC	ATCATGGCCGTCTCTGAAATTC	100	60	0.4
Sfrp1	TACCACGGAAGCCTCTAAGC	GGCCTCTGACTTCAACTCGT	73	60	0.6
Sfrp4	AGCCCTGATCGGTGCAAGT	ATTGCAACCACTCCTCTGGAC	117	60	0.6
Slc32a1	CCATTCAGGGCATGTTCGTG	GAGGAACAACCCCAGGTAGC	71	60	0.6
Syt2	GGCGGCGAGATGTGATACT	GGGTGTCTATGATGGCATCAA	69	60	0.4
Tbp	ACCGTGAATCTTGGCTGTAAAC	GCAGCAAATCGCTTGGGATTA	86	60	0.6
Tfrc	GTTTCTGCCAGCCCCTTATTAT	GCAAGGAAAGGATATGCAGCA	152	60	0.6
Tph2	GCTACCTGAGCCCAAGAGAC	GCATGTATCTGGTTCCGGGG	113	60	0.4
Tuba1a	CCAGGGCTTCTTGGTTTTCC	CTACCATGAAGGCACAATC	218	60	0.6

Ubc	AGCCCAGTGTTACCACCAAG	ACCCAAGAACAAGCACAAGG	97	60	0.6
Vegfa(120aa)	CTTCCTACAGCACAGCAGATG	TGGCTTGTCACATTTTTCTGGC	73	60	0.6
Vegfa(164aa)	AGCACAGCAGATGTGAATGCAG	AGGCTCACAGTGATTTTCTGGC	65	60	0.6
Ywhaz	TGCTGGTGATGACAAGAAAGGA	TGAGGGCCAGACCCAGTCT	119	60	0.4

Table S3. Full gene names.

Abbreviation	Full name
Actb	actin beta
Arnt	aryl hydrocarbon receptor nuclear translocator
B2m	beta-2-microglobulin
Chrm1	cholinergic receptor muscarinic 1
Col1a1	collagen type I alpha 1
Col4a2	collagen type IV alpha 2
Eef2	eukaryotic translation elongation factor 2
Esyt1	extended synaptotagmin 1
Gabbr2	gamma-aminobutyric acid (GABA) B receptor 2
Gabrb1	gamma-aminobutyric acid type A receptor subunit beta1
Gabrd	gamma-aminobutyric acid type A receptor delta subunit
Gapdh	glyceraldehyde-3-phosphate dehydrogenase
Grina	glutamate ionotropic receptor NMDA type subunit associated protein 1
Htr1a	5-hydroxytryptamine receptor 1A
Igf1	insulin like growth factor 1
Nono	non-POU domain containing octamer binding
Nrp1	neuropilin 1
Ogt	O-linked N-acetylglucosamine (GlcNAc) transferase
Parn	poly(A)-specific ribonuclease
Rnh1	ribonuclease inhibitor 1
Sdha	succinate dehydrogenase complex flavoprotein subunit A
Sfrp1	secreted frizzled-related protein 1
Sfrp4	secreted frizzled-related protein 4
Slc32a1	solute carrier family 32 member 1
Syt2	synaptotagmin 2
Tbp	TATA-box binding protein
Tfrc	transferrin receptor
Tph2	tryptophan hydroxylase 2
Tuba1a	tubulin alpha 1A
Ubc	ubiquitin C
Vegf	vascular endothelial growth factor
Ywhaz	tyrosine 3-monooxygenase/tryptophan 5-monooxygenase activation protein zeta

Table S4. Oligonucleotide combinations used for quantitative bisulfite pyrosequencing methylation analysis of *Gabbr2* and *Syt2* genes in the genomic segments described in Figure S3, as well as for *Chrm1*, *Igf1*, *Sfrp1* and *Sfrp4* genes.

Oligonucleotide sequence [a] and orientation [b]		Amplified segment	Amplicon size (bp)
TGGTTTTGTTGGGAGAGATGTAAGA	(S)	Mouse *Gabbr2* promoter region	208
5'Biot- ACCCTAACTACCCACTCTAAAACTA	(A)		
GAGGATAGGAGAGTAGGGGTT	(SEQ1)		
TTTTTGGGTTAGGAAAAATT	(SEQ2)		
5'Biot- AAACCCTAAAACCAAAAACAAACTTCTCTAC	(S)	Mouse *Syt2* promoter region	183
AGGGGTAAAAGAGGTAGGTTG	(A)		
CCACAATATTTATTTAAACAATTC	(SEQ1)		
AAATAGGAGGGGGATGGG	(SEQ2)		
GTTTAGGGAAGGAAAAGTTAGGAATAGAGT	(SEQ3)		
TGTTTTTTTTTTTTGTTGTGAGGGAGTTAAA	(S)	Mouse *Chrm1* promoter region	107
5'-biot-AAACCAACCCTCTAAATCTAATCCAAATTAA	(A)		
TTTGTTTGAAAATGATATATTGG	(SEQ1)		
ATTGGGTAAGTGGTTGGTAGTAT	(S)	Mouse *Igf1* promoter region	174
5'biot-ACCTTCTTTCATAATTCACTTTCTTATCT	(A)		
GTTGGTAGTATGGTTTAAG	(SEQ1)		
GGAGTTTTGATTTTTTGTTGA	(SEQ2)		
ATGGGTAGGTTGAATTTAGGG	(S)	Mouse *Sfrp1* promoter region	161
5'biot-CTAATACCCACATAATCAATACTCTTCT	(A)		
ATTTAGGGAGTTTTTTTAAATT	(SEQ1)		
TGTTTGTGGTTGTGTTTTAGGTGA	(SEQ2)		
GTAGAATTATGGTTTTTGGTTGTGG	(S)	Mouse *Sfrp4* promoter region	137
5'biot-TCCCCTAACCTAAACTCAACAACCTTA	(A)		
TGGAGAGGTAGAGATGATGT	(SEQ1)		
TGTAGGAGAGGTTGGGGGTG	(SEQ2)		

[a] 5'Biot- designates a 5'-biotinylated oligonucleotide.
[b] (S), sense PCR primer; (A), antisense PCR primer; (SEQ1), (SEQ2) and (SEQ3), sequencing primer, same polarity as the non-biotinylated PCR primer.

Table S5. Oligonucleotide primer sets used for ePAT analysis of polyadenylated mRNAs.

Oligonucleotide[(a)]	Sequence	Orientation[(b)]
PAT anchor primer	GCGAGCTCCGCGGCCGCGTTTTTTTTTTTT	(A)
TVN anchor primer	GCGAGCTCCGCGGCCGCGTTTTTTTTTTTTVN	(A)
Eef2 -141 bp	GTGGGGTAAGTGCCTCCAG	(S)
Eef2 - 169 bp	GCCAAGTGGAGTTCCCCCAG	(S)
Eef2 - 172 bp	ATGGCCAAGTGGAGTTCCC	(S)
Eef2 - 176 bp	GTCCATGGCCAAGTGGAGT	(S)
Nono - 113 bp	CAGATACCTTCAGTTTGGAAAGTT	(S)
Nono - 123 bp	TGAATTGTTCCAGATACCTTCAGT	(S)
Nono - 159 bp	TAGGTAGCTACGCTGTGCAT	(S)
Tfrc - 125 bp	TGTCTTTGCTGAAATGCCCTT	(S)
Tfrc - 126 bp	CTGTCTTTGCTGAAATGCCCT	(S)
Tfrc - 127 bp	ACTGTCTTTGCTGAAATGCCC	(S)
Tfrc - 139 bp	TGCCAACCTATACTGTCTTTGCT	(S)

[a] For gene-specific sense primers, the bp number indicates the expected size for the TVN amplicon.
[b] (S), sense; (A), antisense.

Table S6. 15 genes showing a diet effect for both sexes in P30 pups

ID	Gene Symbol	Description	Chromosome	Group
TC0100000637.mm.2	*Plcd4*	phospholipase C, delta 4	chr1	Multiple_Complex
TC0100000640.mm.2	*Ttll4*	tubulin tyrosine ligase-like family, member 4	chr1	Multiple_Complex
TC0200001779.mm.2	*Rtf1*	Rtf1, Paf1/RNA polymerase II complex component, homolog (S. cerevisiae)	chr2	Multiple_Complex
TC0200003177.mm.2	*C330006A16Rik*	RIKEN cDNA C330006A16 gene	chr2	Multiple_Complex
TC0300000841.mm.2	*Gm4858*	predicted gene 4858	chr3	Coding
TC0300001362.mm.2	*Tacr3*	tachykinin receptor 3	chr3	Coding
TC0600000409.mm.2	*Wee2*	WEE1 homolog 2 (S. pombe)	chr6	Coding
TC0600002743.mm.2	*Trh*	thyrotropin releasing hormone	chr6	Coding
TC0700001409.mm.2	*Ucp2*	uncoupling protein 2 (mitochondrial, proton carrier)	chr7	Multiple_Complex
TC0X00001955.mm.2	*Klhl13*	kelch-like 13	chrX	Multiple_Complex
TC0X00002274.mm.2	*Gpr101*	G protein-coupled receptor 101	chrX	Multiple_Complex
TC1200001912.mm.2	*Six1*	sine oculis-related homeobox 1	chr12	Multiple_Complex
TC1500000346.mm.2	*Trhr*	thyrotropin releasing hormone receptor	chr15	Coding
TC1600000508.mm.2	*Gm5483*	predicted gene 5483	chr16	Multiple_Complex
TC1600000741.mm.2	*Senp7*	SUMO1/sentrin specific peptidase 7	chr16	Multiple_Complex

Table S7. 23 genes showing sex differences for both diets in P30 pups.

ID	Gene Symbol	Description	Chromosome	Group
TC0100000637.mm.2	*Plcd4*	phospholipase C, delta 4	chr1	Multiple_Complex
TC0100000640.mm.2	*Ttll4*	tubulin tyrosine ligase-like family, member 4	chr1	Multiple_Complex
TC0200001340.mm.2	*Olfr1261*	olfactory receptor 1261	chr2	Coding
TC0400001715.mm.2	*Eif4g3*	eukaryotic translation initiation factor 4 gamma, 3	chr4	Multiple_Complex
TC0400004191.mm.2	*Slc6a9*	solute carrier family 6 (neurotransmitter transporter, glycine), member 9	chr4	Multiple_Complex
TC0500001205.mm.2	*Wscd2*	WSC domain containing 2	chr5	Multiple_Complex
TC0600002714.mm.2	*Cfap100*	cilia and flagella associated protein 100	chr6	Multiple_Complex
TC0700001409.mm.2	*Ucp2*	uncoupling protein 2 (mitochondrial, proton carrier)	chr7	Multiple_Complex
TC0700002178.mm.2	*Sbk2*	SH3-binding domain kinase family, member 2	chr7	Multiple_Complex
TC0700002373.mm.2	*Sult2a4*	sulfotransferase family 2A, dehydroepiandrosterone (DHEA)-preferring, member 4	chr7	Coding
TC0X00000190.mm.2	*Kdm6a*	lysine (K)-specific demethylase 6A	chrX	Multiple_Complex
TC0X00002643.mm.2	*Eif2s3x*	eukaryotic translation initiation factor 2, subunit 3, structural gene X-linked	chrX	Multiple_Complex
TC0Y00000005.mm.2	*Kdm5d*	lysine (K)-specific demethylase 5D	chrY	Multiple_Complex
TC0Y00000006.mm.2	*Eif2s3y*	eukaryotic translation initiation factor 2, subunit 3, structural gene Y-linked	chrY	Multiple_Complex
TC0Y00000066.mm.2	*Gm21768*	predicted gene, 21768	chrY	Coding
TC0Y00000233.mm.2	*Uty*	ubiquitously transcribed tetratricopeptide repeat gene, Y chromosome	chrY	Multiple_Complex
TC0Y00000235.mm.2	*Ddx3y*	DEAD (Asp-Glu-Ala-Asp) box polypeptide 3, Y-linked	chrY	Multiple_Complex
TC1100002004.mm.2	*Endov*	endonuclease V	chr11	Multiple_Complex
TC1200001912.mm.2	*Six1*	sine oculis-related homeobox 1	chr12	Multiple_Complex
TC1300000020.mm.2	*Akr1c19*	aldo-keto reductase family 1, member C19	chr13	Multiple_Complex

TC1600001788.mm.2	*Olfr190*	olfactory receptor 190	chr16	Coding
TC1900000467.mm.2	*Papss2*	3-phosphoadenosine 5-phosphosulfate synthase 2; 3'-phosphoadenosine 5'-phosphosulfate synthase 2	chr19	Multiple_Complex
TC1900000928.mm.2	*Gm960*	predicted gene 960	chr19	Coding

Table S8. Five genes showing a diet effect for both sexes in E17.5 embryos.

ID	Gene Symbol	Description	Chromosome	Group
TC0200001276.mm.2	*Olfr1112*	olfactory receptor 1112	chr2	Coding
TC0400002381.mm.2	*Spaca1*	sperm acrosome associated 1	chr4	Coding
TC0X00002031.mm.2	*Gm2964*	predicted gene 2964 [Source:MGI Symbol;Acc:MGI:3781142]; predicted gene 2964	chrX	Coding
TC0Y00000067.mm.2	*Gm20840*	predicted gene, 20840	chrY	Coding
TC1100001293.mm.2	*Bcas3*	breast carcinoma amplified sequence 3	chr11	Multiple_Complex

Table S9. Three genes showing a diet effect for both P30 and E17.5 males.

ID	Gene Symbol	Description	Chromosome	Group
TC0X00002805.mm.2	*Magee2*	melanoma antigen, family E, 2	chrX	Coding
TC1000003019.mm.2	*Irak3*	interleukin-1 receptor-associated kinase 3	chr10	Multiple_Complex
TC1100001848.mm.2	*Kcnj16*	potassium inwardly-rectifying channel, subfamily J, member 16	chr11	Coding

Table S10. 12 genes showing a diet effect in both P30 and E17.5 females.

ID	Gene Symbol	Description	Chromosome	Group
TC0200003300.mm.2	*Dnm1*	dynamin 1	chr2	Multiple_Complex
TC0200003747.mm.2	*Gm11084*	predicted gene 11084 [Source:MGI Symbol;Acc:MGI:3779315]	chr2	Coding
TC0400000982.mm.2	*Inadl*	InaD-like (Drosophila)	chr4	Multiple_Complex
TC0500000947.mm.2	*Prdm8*	PR domain containing 8	chr5	Multiple_Complex
TC0600000014.mm.2	*Peg10*	paternally expressed 10	chr6	Coding
TC0900000151.mm.2	*Olfr851*	olfactory receptor 851	chr9	Coding
TC1100001251.mm.2	*1700020L24Rik*	RIKEN cDNA 1700020L24 gene	chr11	Coding
TC1100001448.mm.2	*Col1a1*	collagen, type I, alpha 1	chr11	Multiple_Complex
TC1600000288.mm.2	*Eif4g1*	eukaryotic translation initiation factor 4, gamma 1	chr16	Multiple_Complex
TC1600002145.mm.2	*Dscam*	Down syndrome cell adhesion molecule	chr16	Coding
TC1700000576.mm.2	*LOC100861596*	PREDICTED: zinc finger protein 791-like (LOC100861596), mRNA.	chr17	Coding
TSUnmapped00000005.mm.2	*Amot*	angiomotin	chrX	Coding

Table S11. 15 genes showing sex differences for both diets in E17.5 embryos.

ID	Gene Symbol	Description	Chromosome	Group
TC0200002550.mm.2	*Eya2*	eyes absent 2 homolog (Drosophila)	chr2	Multiple_Complex
TC0500000690.mm.2	*Spata18*	spermatogenesis associated 18	chr5	Coding
TC0500003743.mm.2	*Psmg3*	proteasome (prosome, macropain) assembly chaperone 3	chr5	Multiple_Complex
TC0600000409.mm.2	*Wee2*	WEE1 homolog 2 (S. pombe)	chr6	Coding
TC0600002413.mm.2	*Vmn1r33*	vomeronasal 1 receptor 33	chr6	Coding
TC0900000679.mm.2	*Pstpip1*	proline-serine-threonine phosphatase-interacting protein 1	chr9	Multiple_Complex
TC0900002044.mm.2	*Slc37a2*	solute carrier family 37 (glycerol-3-phosphate transporter), member 2	chr9	Multiple_Complex
TC0X00000190.mm.2	*Kdm6a*	lysine (K)-specific demethylase 6A	chrX	Multiple_Complex
TC0Y00000005.mm.2	*Kdm5d*	lysine (K)-specific demethylase 5D	chrY	Multiple_Complex
TC0Y00000006.mm.2	*Eif2s3y*	eukaryotic translation initiation factor 2, subunit 3, structural gene Y-linked	chrY	Multiple_Complex
TC0Y00000233.mm.2	*Uty*	ubiquitously transcribed tetratricopeptide repeat gene, Y chromosome	chrY	Multiple_Complex
TC0Y00000235.mm.2	*Ddx3y*	DEAD (Asp-Glu-Ala-Asp) box polypeptide 3, Y-linked	chrY	Multiple_Complex
TC1100002084.mm.2	*Limk2*	LIM motif-containing protein kinase 2	chr11	Multiple_Complex
TC1200000716.mm.2	*Rps26*	ribosomal protein S26	chr12	Multiple_Complex
TC1900000026.mm.2	*Cdk2ap2*	CDK2-associated protein 2	chr19	Multiple_Complex
